# Real-world short-term effectiveness of erenumab after single versus multiple prior preventive treatment failures

**DOI:** 10.1186/s12883-026-05311-8

**Published:** 2026-08-28

**Authors:** Chantal Schmidt, Lukas Becker, Diana Lindner, Christoph Kleinschnitz, Alexander Wolfgang Becker, Dagny Holle, Armin Scheffler

**Affiliations:** 1 Department of Neurology and Center for Translational Neuro- and Behavioral Sciences (C-TNBS), West German Headache Center, University Hospital Essen, University Duisburg-Essen, Essen, Germany; 2 Medical Faculty, Institute for General Practice/Family Medicine, University of Duisburg-Essen, Essen, Germany; 3 Department of Psychiatry and Psychotherapy, LVR-University Hospital Essen, University Duisburg-Essen, Essen, Germany

**Keywords:** CGRP, Migraine, Chronification, Monoclonal antibody

## Abstract

**Background:**

Erenumab, a monoclonal antibody targeting calcitonin gene-related peptide receptor, has been approved in Germany since 2018 for migraine prophylaxis. Initially, reimbursement regulations restricted its use to drug-resistant patients who had failed or were unsuitable for conventional preventive treatments. Over time, initiation after failure of only one prior preventive treatment became possible. As earlier initiation of effective preventive therapy may improve clinical outcomes, we compared the effectiveness of erenumab in patients with single versus multiple prior preventive treatment failures.

**Methods:**

In this retrospective single-center study, data from 196 patients with episodic or chronic migraine who received erenumab for at least three months were analysed. Patients were categorised into a multiple prior preventive therapy (MPT) group (*n* = 140), comprising individuals with two to six previous preventive treatment failures, and single preventive therapy (SPT) group (*n* = 56), including patients who had received no more than one prior treatment failure before initiating erenumab. To evaluate the association between prior treatment exposure and treatment response, a multivariate logistic regression analysis was performed with achievement of a ≥ 50% reduction in monthly headache days (MHD) as the primary outcome. Subgroup analyses, LASSO-based variable selection, and propensity score weighting were conducted as sensitivity analyses.

**Results:**

A clinically meaningful treatment response (50% MHD response) was achieved by 35.7% (*n* = 20/56) of patients in the SPT group and 30.7% (*n* = 43/140) in the MPT group. No significant difference in treatment effectiveness was observed between groups. In the adjusted logistic regression model, SPT was not significantly associated with treatment response (SPT vs. MPT: adjusted OR 1.39, 95% CI 0.53–3.69; *p* = 0.5).

**Conclusion:**

Short-term effectiveness of erenumab was comparable in patients with no more than one versus multiple prior preventive treatment failures, suggesting that treatment response is not solely dependent on the number of previous preventive treatment failures. These findings should be interpreted in light of the retrospective single-center design and the limited sample size, which resulted in relatively wide confidence intervals.

**Supplementary Information:**

The online version contains supplementary material available at https://doi.org/10.1186/s12883-026-05311-8.

## Background

Monoclonal antibodies (mAb) targeting the calcitonin-gene-related peptide (CGRP) pathway, either by binding to the peptide itself or its receptor, have become an essential part of the prophylactic treatment of migraine. Erenumab is a CGRP mAb, which selectively binds the CGRP receptor and modulates the initiation and maintenance of migraine [1]. It was approved by the European Medicines Agency (EMA) in July 2018 for the prevention of migraine in adults experiencing at least four migraine days per month [1] and showed good efficacy and tolerability, both in short-term [2] and in the long-term [3, 4] treatment in open-label and real-world studies.

Due to reimbursement regulations at the time of its approval, erenumab was initially available in Germany only to patients who had failed all non-specific preventive treatments (amitriptyline, metoprolol/propranolol, topiramate, flunarizine, onabotulinumtoxinA), were not eligible for them, or had experienced adverse events from these medications [5]. After the HER-MES trial demonstrated the superiority of erenumab over topiramate in its primary endpoint [6], reimbursement policies were subsequently revised [5]. Since then, only a single prior preventive treatment has been required for reimbursement by statutory health insurance, and this approach has also been incorporated into the German clinical guidelines [7]. The change in reimbursement policy expanded access to erenumab and allowed its use at an earlier stage of the treatment pathway.

It has not been conclusively determined whether the timing of treatment has a relevant impact on therapeutic outcomes. Despite promising long-term results [3, 4], it remains uncertain whether initiation of CGRP mAbs at the beginning of the medical treatment leads to improved outcomes. Likewise, it is unclear whether initiation as the last therapy option diminishes therapeutic effectiveness. Although limited real-world data suggest that use as an early preventive drug may be associated with more favourable outcomes [5], evidence remains insufficient.

To provide further evidence on this clinically relevant question, we analysed the short-term clinical response to erenumab in relation to treatment timing, examining whether the drug was initiated early or late within the therapeutic pathway.

## Methods

A retrospective analysis of routine clinical data from migraine patients was performed. All patients presented at the West German Headache Center in Essen between 2018 and 2024 and received erenumab for at least 3 months. The analysis was approved by the independent ethics committee of University Hospital Essen (19-9004-BO). Given this was a retrospective analysis of internal routine data, written informed consent was not needed. A subset of the data from this patient cohort has previously been published in the context of a different research question addressing the overall short-term effectiveness of erenumab in therapy-refractory patients [2].

We classified patients as having either episodic migraine (EM) or chronic migraine (CM) according to the ICHD-3 criteria [8]. CM patients who had overused acute headache medication for at least three months, defined as intake on more than 10 or 15 days per month depending on the medication type, were classified as having medication overuse headache (MOH) [8].

Patients were divided into two groups based on their prior therapy regime. Multiple prior preventive therapies (MPT) was defined as initiation of erenumab after at least two and up to six prior preventive treatment failures. Prior preventive therapies included guideline-recommended agents such as beta-blockers, topiramate, flunarizine, amitriptyline, and onabotulinumtoxinA (for patients with CM). Until 2020, valproic acid was considered a standard preventive treatment option. Consequently, patients could have failed up to six preventive medications before initiating CGRP-targeting therapy. Following its removal from the standard treatment recommendations, prior treatment with valproic acid was no longer required, reducing the number of prior preventive treatment failures required before initiation of CGRP-targeting therapy to five. Single prior preventive therapy (SPT) was defined as initiation of erenumab after no more than one prior treatment failure among the preventive therapies listed above.

Patients were excluded if baseline or 3-month follow-up data were completely missing or insufficiently documented, if they had fewer than four monthly migraine days (MMD) at treatment initiation, if they had previously been treated with other CGRP mAbs, if they had participated in clinical trials involving CGRP-targeted therapies (including gepants), or if they had received erenumab prior to treatment at our center. Continuation of an ongoing preventive therapy was allowed, as long as it had not been newly initiated at the beginning of the observation period.

A standardised questionnaire, headache diaries and medical documentation were used to assess monthly headache days (MHD), MMD and days with acute medication use (AMD). Where available, headache diary-based data were used for outcome assessment; otherwise, retrospectively collected data from standardised questionnaires and medical records were utilised. An English-translated version of the questionnaires before and after three months of therapy can be found in the Supplementary Material (see Supplementary Material Q0mo and Q3mo).

Baseline demographic and clinical characteristics were compared between patients with SPT and MPT. Continuous variables are presented as mean (standard deviation) or median (interquartile range), as appropriate, and categorical variables as frequencies and percentages. Between-group differences were assessed using Student’s t-test or Wilcoxon rank-sum test for continuous variables, and Pearson’s chi-square test or Fisher’s exact test for categorical variables, as appropriate.

Baseline and 3-month outcomes were compared between the two treatment groups. Continuous variables were summarised as median and interquartile range (IQR). Within-group changes from baseline to month 3 for MHD, MMD and AMD were assessed using the paired Wilcoxon signed-rank test. To account for multiple testing, Bonferroni correction was applied, resulting in a significance level of α = 0.017. Between-group differences in treatment response were assessed using the Mann-Whitney U test. Effect sizes for continuous outcomes were reported as Hodges-Lehmann estimates with corresponding 95% confidence intervals (CIs). Responder rates (30%, 50%, and 75% reduction from baseline in MMD, MHD, and AMD) were compared between groups using univariate logistic regression models. Odds ratios (ORs) with 95% CIs were calculated, with MPT as the reference group. To account for potential confounding, an adjusted multivariate logistic regression model was performed to estimate the association between treatment group (SPT vs. MPT) and treatment response (≥ 50% reduction in MHD), while adjusting a priori for age, sex, baseline MHD, chronic migraine, daily headache, MOH, psychiatric comorbidity, concomitant preventive medication, source of data collection (headache diary or medical documentation), and baseline erenumab dose (70 mg vs. 140 mg). Multicollinearity among covariates was assessed using variance inflation factors (VIFs), with values < 5 considered indicative of the absence of problematic multicollinearity. Migraine duration and aura status were not included in the primary model because of missing data but were examined in a complete-case sensitivity analysis. Adjusted ORs with 95% CIs and corresponding p-values were calculated.

To evaluate whether modelling previous preventive treatment exposure as a continuous variable affected the results, an additional multivariate logistic regression model replacing treatment group with the number of previous preventive treatment failures was performed.

As exploratory analyses, LASSO-penalised logistic regression with 10-fold cross-validation was performed to identify candidate predictors of treatment response. Treatment group was retained in all models irrespective of penalized variable selection because it represented the primary exposure of interest.

As a further sensitivity analysis, inverse probability of treatment weighting (IPTW) based on the propensity score was performed to estimate the average treatment effect (ATE) and reduce baseline imbalance between treatment groups. Stabilised inverse probability weights were applied, and covariate balance was assessed using absolute standardized mean differences (SMDs), with values < 0.10 considered indicative of adequate balance. Covariates with residual imbalance after weighting (absolute SMD ≥ 0.10) were additionally included in the weighted outcome model, resulting in a doubly robust estimation approach.

Tolerability and adverse events were evaluated descriptively and compared using Fisher’s exact test. Detailed migraine characteristics, including attack duration, attack intensity, response to acute medication, and accompanying symptoms (photophobia, phonophobia, nausea, dizziness, need for rest, and aura), were assessed descriptively.

Statistical analyses were performed using R 4.5.2 (R Foundation for Statistical Computing, Vienna, Austria). Most figures were created in R, with final figure preparation performed using Microsoft Office 365 (Version 2506, Microsoft Corporation, Redmond, WA, USA).

## Results

**Fig. 1:**
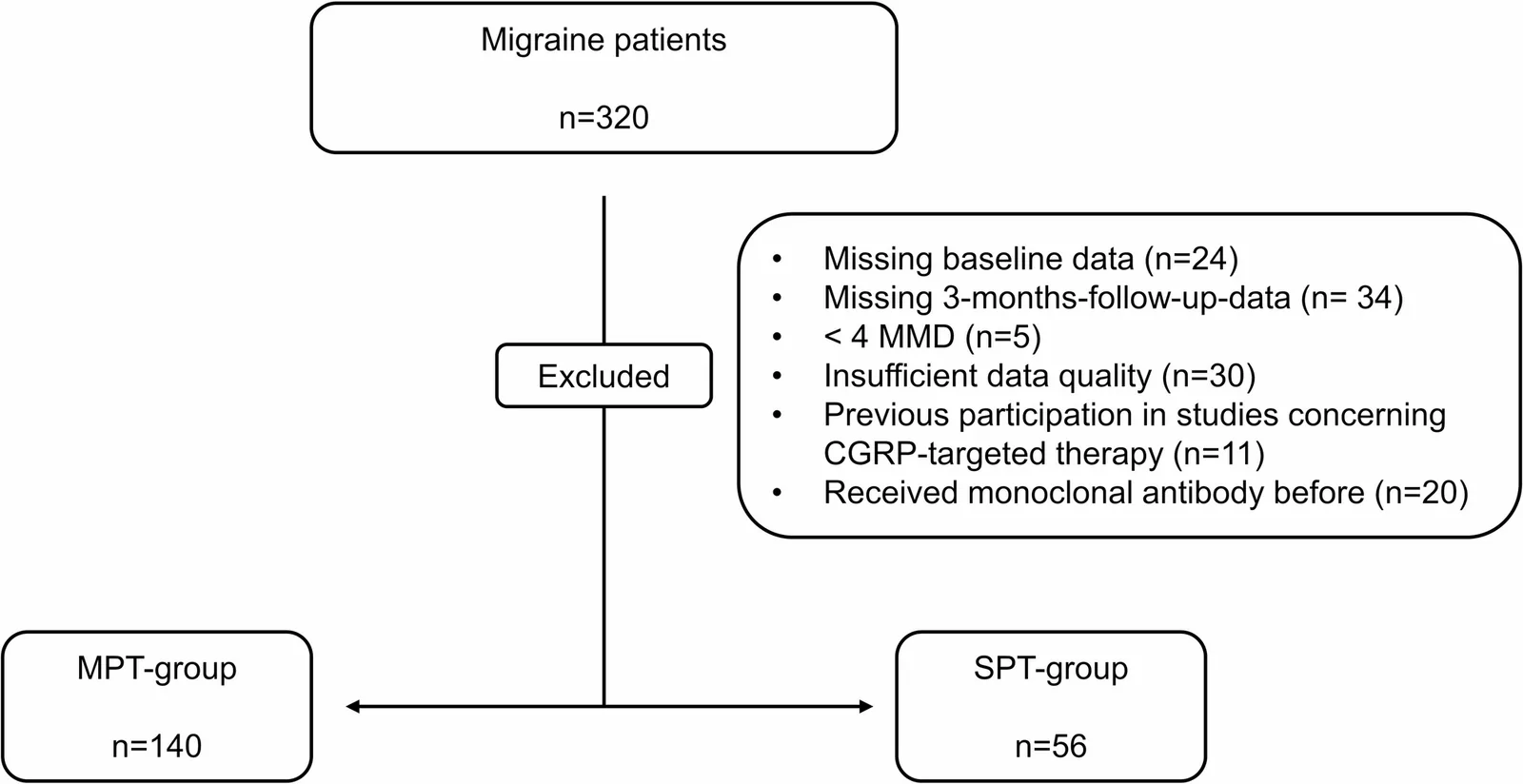
Overview of patient selection. (Abbreviations: SPT: Single prior preventive therapy, MPT: Multiple prior preventive therapies, MMD: monthly migraine days)

Overall, 320 patients received erenumab treatment. After application of the predefined exclusion criteria, 196 patients were included in the final analysis (Fig. 1). One patient younger than 18 years received erenumab as off-label treatment.

Baseline characteristics are summarised in Table 1. Patients in the MPT group were significantly older, had a higher baseline headache frequency, and more frequently fulfilled the criteria for chronic migraine and MOH than patients in the SPT group. Concomitant preventive medication was more common in the MPT group, whereas patients in the SPT group more frequently received an initial erenumab dose of 140 mg. Migraine duration was also longer in the MPT group. No significant differences were observed regarding sex, daily headache, psychiatric comorbidity, or migraine aura. Baseline MHD, MMD and AMD values stratified by treatment group are shown as boxplots in Supplementary Figure S1.

**Table 1:** Characteristics of a cohort of 196 migraine patients treated with erenumab in the West German Headache Center

	MPT (*N*=140)	SPT (*N*=56)	Total (*N*=196)	*p* value
Age				0.047
Median (IQR)	49.0 (38.0 - 56.0)	42.5 (32.8 - 54.0)	48.0 (37.0 - 55.2)	
Sex				0.528
Male	36 (25.7%)	12 (21.4%)	48 (24.5%)	
Female	104 (74.3%)	44 (78.6%)	148 (75.5%)	
Baseline MHD				0.048
Median (IQR)	17.0 (13.7 - 25.0)	13.3 (9.4 - 22.0)	15.7 (12.0 - 25.0)	
Chronic migraine				< 0.001
yes	101 (72.1%)	26 (46.4%)	127 (64.8%)	
Daily headache				0.555
yes	18 (12.9%)	9 (16.1%)	27 (13.8%)	
MOH				0.020
	60 (42.9%)	14 (25.0%)	74 (37.8%)	
Psychiatric disorders				0.127
	24 (17.1%)	15 (26.8%)	39 (19.9%)	
Additional conventional comedication				0.011
	68 (48.6%)	16 (28.6%)	84 (42.9%)	
Erenumab dose				< 0.001
70mg	123 (87.9%)	13 (23.2%)	136 (69.4%)	
140mg	17 (12.1%)	43 (76.8%)	60 (30.6%)	
Migraine Duration in Years				0.003
Median (IQR)	30.0 (19.0 - 39.0)	23.5 (11.8 - 31.0)	27.0 (16.2 - 37.0)	
Aura				0.174
yes	57 (40.7%)	31 (55.4%)	88 (44.9%)	
NA	4 (2.9%)	1 (1.8%)	5 (2.6%)	
Documentation				< 0.001
Headache Diaries	101 (72.1%)	11 (19.6%)	112 (57.1%)	
Medical documentation	39 (27.9%)	45 (80.4%)	84 (42.9%)	
Number of prior preventives				
Median (IQR)	5.0 (4-5.25)	1 (1-1)	4 (1-5)	< 0.001
Year of treatment initiation				< 0.001
2018	34 (24.2%)	0 (0%)	34 (17.3%)	
2019	66 (47.1%)	2 (3.6%)	68 (34.7%)	
2020	19 (13.6%)	1 (1.8%)	20 (10.2%)	
2021	17 (12.1%)	9 (16.1%)	26 (13.3%)	
2022	4 (2.9%)	9 (16.1%)	13 (6.6%)	
2023	0 (0%)	35 (62.5%)	35 (17.9%)	

Comorbid psychiatric disorders including depression, anxiety, bipolar disorders

*Abbreviations*: *SPT* Single prior preventive therapy, *MPT* Multiple prior preventive therapies, *MHD* Monthly headache days, *MOH* Medication overuse headache, *IQR* Interquartile range

**Fig. 2:**
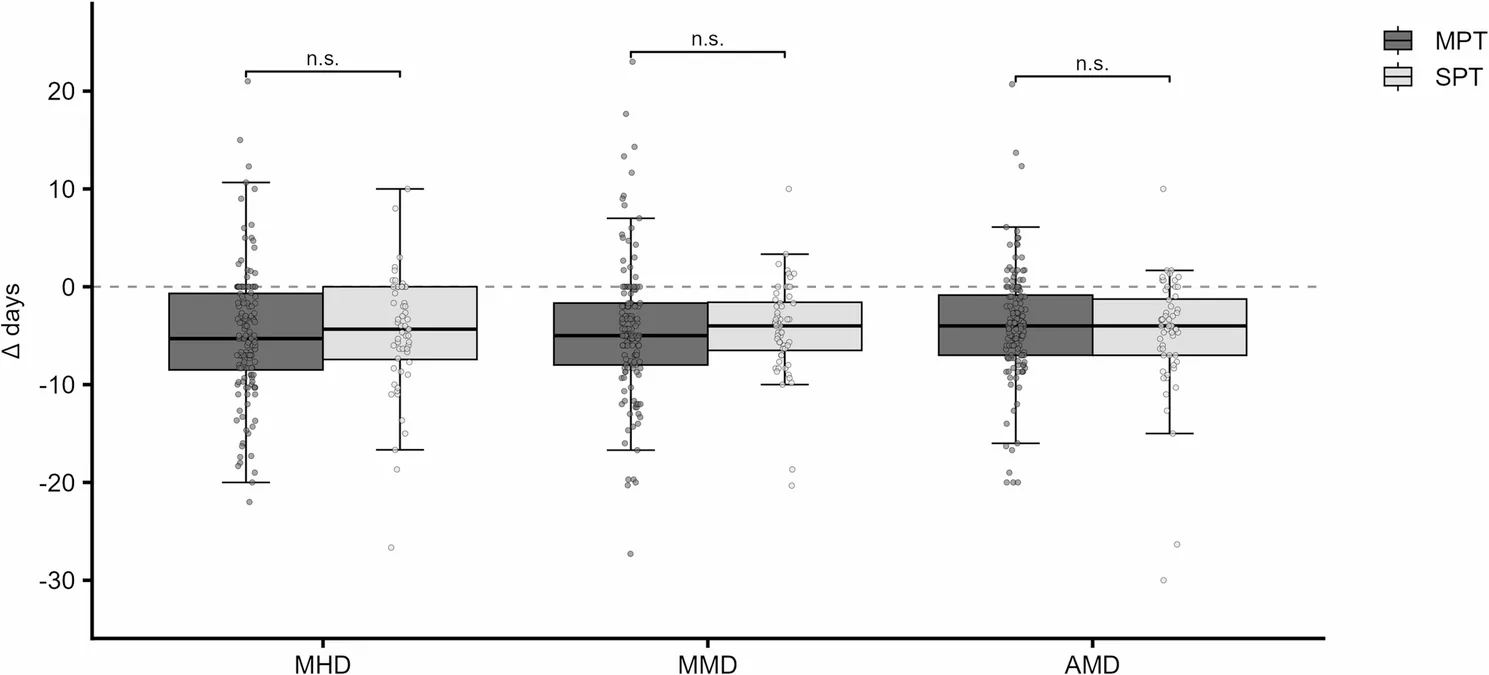
Change in MHD, MMD and AMD of SPT and MPT group after 3 months of therapy. Group comparisons were performed using the Mann-Whitney U test, significance levels are indicated. (Abbreviations: AMD: Acute medication days, MPT: Multiple prior preventive therapies, MHD: Monthly headache days, MMD: Monthly migraine days, n.s.: Non-significant, SPT: Single prior preventive therapy.)

After three months of erenumab treatment, MHD, MMD, and AMD were significantly reduced in both the SPT and MPT group (Table 2). Analysis using the Mann-Whitney U test showed no significant between-group differences in the reduction of MHD, MMD, or AMD after three months of treatment. When stratified by migraine type, both groups demonstrated significant reductions in MHD, MMD, and AMD among patients with EM as well as CM. No statistically significant differences in reduction from baseline were observed between the groups (Fig. 2).

**Table 2:** Baseline- and 3-months values for MMD, MHD and AMD

	Episodic Migraine				Chronic Migraine				Overall			
	MPT (*n*=39)	SPT (*n*= 30)	Between-group difference (95% CI)**	*p*-value****	MPT (*n*=101)	SPT (*n*=26)	Between-group difference (95% CI)**	*p*-value****	MPT (*n*=140)	SPT (*n*=56)	Between-group difference (95% CI)**	*p*-value****
MHD, median (IQR)												
Baseline	10.3 (8.5-12.0)	9.8 (7.5-11.5)	1.0 (-0.0–2.3)	0.109	20.0 (15.3-26.7)	25.8 (18.3-30.0)	-1.7 (-5.0–0.0)	0.070	17.0 (13.7-25.0)	13.3 (9.4-22.0)	2.7 (0.0–5.0)	0.019
After 3 months	6.0 (3.8-8.7)	4.5 (3.7-8.5)	1.3 (-0.7–3.0)	0.193	14.7 (9.3-23.0)	17.0 (9.7-28.8)	-1.6 (-6.0–2.0)	0.316	11.7 (6.2-18.8)	8.5 (4.0-15.3)	2.3 (0.0–4.7)	0.045
Within-group reduction (95% CI)*	4.2 (3.0 to 5.1)	4.2 (2.7 to 5.3)	0.0 (-1.7 to 2.0)	0.865	7.0 (5.5 to 8.5)	7.3 (3.8 to 11.0)	0.0 (-3.3 to 3.0)	0.909	5.9 (4.8 to 6.8)	5.2 (3.8 to 6.7)	0.7 (-1.0 to 2.3)	0.486
*p*-value***	<0.001	<0.001			<0.001	0.001			<0.001	<0.001		
MMD, median (IQR)												
Baseline	10.0 (8.0-11.3)	7.7 (6.2-10.0)	1.6 (0.0–3.0)	0.025	15.0 (10.0-18.0)	13.3 (10.0-19.0)	0.3 (-2.0–3.7)	0.593	12.3 (10.0-15.0)	10.0 (6.7-12.8)	2.7 (1.0–4.3)	<0.001
After 3 months	5.0 (3.0-7.3)	3.7 (2.4-5.2)	1.3 (-0.3–2.7)	0.103	7.7 (4.3-14.7)	6.7 (5.0-14.6)	0.0 (-2.3–2.8)	0.869	6.8 (4.0-12.0)	4.8 (3.2-7.5)	1.7 (0.3–3.3)	0.015
Within-group reduction (95% CI)*	4.8 (3.7 to 6.0)	4.3 (3.3 to 5.7)	0.3 (-1.4 to 1.9)	0.795	5.2 (3.9 to 6.7)	4.8 (2.7 to 7.2)	0.7 (-2.0 to 3.3)	0.658	5.1 (4.2 to 6.0)	4.5 (3.5 to 5.7)	0.3 (-1.0 to 2.0)	0.553
*p*-value***	<0.001	<0.001			<0.001	0.002			<0.001	<0.001		
AMD, median (IQR)												
Baseline	9.0 (6.5-10.0)	8.0 (6.0-11.0)	0.3 (-1.3–2.0)	0.720	10.0 (8.0-15.0)	10.0 (3.5-13.1)	2.3 (-0.7–5.0)	0.149	10.0 (8.0-14.0)	8.0 (4.8-11.7)	2.0 (0.0–3.3)	0.035
After 3 months	4.6 (3.1-6.5)	3.7 (1.7-5.0)	1.0 (-0.3–2.3)	0.101	7.3 (4.0-10.0)	4.2 (1.9-5.9)	2.7 (0.7–4.3)	0.004	6.0 (3.7-9.7)	3.8 (1.7-5.7)	2.3 (1.0–3.3)	<0.001
Within-group reduction (95% CI)*	3.9 (2.6 to 5.0)	4.5 (3.0 to 6.3)	-0.5 (-2.7 to 1.3)	0.590	4.3 (3.2 to 5.3)	4.3 (2.7 to 6.7)	-0.3 (-2.6 to 1.7)	0.691	4.1 (3.3 to 5.0)	4.5 (3.3 to 5.8)	-0.3 (-1.7 to 1.0)	0.619
*p*-value***	<0.001	<0.001			<0.001	<0.001			<0.001	<0.001		

Within-group changes were assessed using the paired Wilcoxon signed-rank test, and between-group comparisons were performed using the Mann-Whitney U test. Effect sizes are reported as Hodges-Lehmann estimates with corresponding 95% confidence intervals

*Abbreviations*: *AMD* Acute medication days, *CI* Confidence interval, *IQR* Interquartile range, *MPT* Multiple prior preventive therapies, *MHD* Monthly headache days, *MMD* Monthly migraine days, *SPT* Single prior preventive therapy

* Within-group changes are presented as the Hodges-Lehmann estimate of the paired difference (baseline value minus Month 3 value) together with the corresponding 95% confidence interval

** Between-group differences were estimated using the Hodges-Lehmann estimator with 95% confidence intervals (Mann-Whitney U test). Estimates represent the median of all pairwise differences between groups and may differ from the difference between group medians

*** Paired Wilcoxon signed-rank test

**** Mann-Whitney U test

Overall, 32% (*n* = 63/196) of patients had a ≥ 50% reduction in MHD after three months of treatment. All response rates are summarised in Table 3. Responder rates were comparable between treatment groups, with 36% (*n* = 20/56) of patients in the SPT group and 31% (*n* = 43/140) in the MPT group achieving the primary endpoint. In the unadjusted analysis, treatment group was not associated with treatment response (OR: 1.25, CI: 0.65–2.41, *p* = 0.503).

**Table 3:** Responder rates (30%, 50% and 75%) for MHD, MMD and AMD

		Episodic			Chronic			All		
	Response	MPT	SPT	OR (95% CI), p	MPT	SPT	OR (95% CI), p	MPT	SPT	OR (95% CI), p
MHD	≥30%	24/39 (61.5%)	21/30 (70%)	1.46 (0.53–4.02), *p* = 0.611	51/101 (50.5%)	12/26 (46.2%)	0.84 (0.35–1.99), *p* = 0.826	75/140 (53.6%)	33/56 (58.9%)	1.24 (0.66–2.33), *p* = 0.528
	≥50%	15/39 (38.5%)	13/30 (43.3%)	1.22 (0.46–3.22), *p* = 0.806	28/101 (27.7%)	7/26 (26.9%)	0.96 (0.36–2.53), *p* = 1	43/140 (30.7%)	20/56 (35.7%)	1.25 (0.65–2.41), *p* = 0.503
	≥75%	2/39 (5.1%)	3/30 (10%)	2.06 (0.32–13.16), *p* = 0.646	7/101 (6.9%)	2/26 (7.7%)	1.12 (0.22–5.74), *p* = 1	9/140 (6.4%)	5/56 (8.9%)	1.43 (0.46–4.46), *p* = 0.547
MMD	≥30%	28/39 (71.8%)	24/30 (80%)	1.57 (0.51–4.89), *p* = 0.575	57/101 (56.4%)	16/26 (61.5%)	1.24 (0.51–2.99), *p* = 0.664	85/140 (60.7%)	40/56 (71.4%)	1.62 (0.83–3.17), *p* = 0.189
	≥50%	22/39 (56.4%)	18/30 (60%)	1.16 (0.44–3.05), *p* = 0.81	42/101 (41.6%)	10/26 (38.5%)	0.88 (0.36–2.12), *p* = 0.826	64/140 (45.7%)	28/56 (50%)	1.19 (0.64–2.21), *p* = 0.636
	≥75%	7/39 (17.9%)	6/30 (20%)	1.14 (0.34–3.84), *p* = 1	17/101 (16.8%)	3/26 (11.5%)	0.64 (0.17–2.39), *p* = 0.763	24/140 (17.1%)	9/56 (16.1%)	0.93 (0.4–2.14), *p* = 1
AMD	≥30%	26/39 (66.7%)	20/30 (66.7%)	1 (0.36–2.74), *p* = 1	58/100* (58%)	19/24** (79.2%)	2.75 (0.95–7.96), *p* = 0.064	84/139 (60.4%)	39/54 (72.2%)	1.7 (0.86–3.38), *p* = 0.137
	≥50%	18/39 (46.2%)	14/30 (46.7%)	1.02 (0.39–2.65), *p* = 1	38/100* (38%)	13/24** (54.2%)	1.93 (0.78–4.74), *p* = 0.17	56/139 (40.3%)	27/54 (50%)	1.48 (0.79–2.79), *p* = 0.258
	≥75%	6/39 (15.4%)	10/30 (33.3%)	2.75 (0.87–8.72), *p* = 0.093	13/100* (13%)	4/24** (16.7%)	1.34 (0.39–4.54), *p* = 0.741	19/139 (13.7%)	14/54 (25.9%)	2.21 (1.02–4.81), *p* = 0.055

Associations with responder status were assessed using univariate logistic regression models and are reported as odds ratios (ORs) with corresponding 95% confidence intervals

*Abbreviations*: *AMD* Acute medication days, *CI* Confidence interval, *MPT* Multiple prior preventive therapies, *MHD* Monthly headache days, *MMD* Monthly migraine days, *SPT* single prior preventive therapy

* One patient with 0 acute medication days (AMD) at baseline

** Two patients with 0 acute medication days (AMD) at baseline

The primary multivariate logistic regression included treatment group (SPT vs. MPT) together with prespecified baseline covariates. The responder rates, defined as a ≥ 50% reduction in MHD, in the population included in this analysis were identical to those observed in the overall cohort, as reported in Table 3 (MPT: 43/140 (30.7%); SPT: 20/56 (35.7%)). As all variables were entered as either binary or continuous predictors, each contributed one parameter to the model, resulting in a total of 11 estimated predictor parameters. The number of events was defined by the number of responders (≥ 50% reduction in MHD) in the overall cohort, comprising 43 of 140 patients in the MPT group and 20 of 56 patients in the SPT group (63 events in total). Accordingly, the events-per-variable (EPV) ratio was calculated as the total number of events divided by the number of predictor parameters (63/11), resulting in an EPV of 5.7. After adjustment, treatment group was not independently associated with achieving a ≥ 50% reduction in MHD (OR 1.39, 95% CI 0.53–3.69; *p* = 0.5). Likewise, age, sex, baseline headache frequency, psychiatric comorbidity, concomitant preventive medication, and baseline erenumab dose were not significantly associated with treatment response. Patients with MOH showed significantly higher odds of achieving treatment response (OR 4.09, 95% CI 1.55–12.3; *p* = 0.007), whereas chronic migraine showed a non-significant trend towards lower response rates. The adjusted model is presented in Table 4. Assessment of multicollinearity demonstrated no evidence of problematic collinearity among the included covariates (all variance inflation factors < 5).

**Table 4:** Results of the multivariate logistic regression analysis with ≥ 50% MHD response as the dependent variable

Characteristic	OR^1^	95% CI^1^	*p*-value
Treatment group (SPT vs. MPT)			
MPT	—	—	
SPT	1.39	0.53, 3.69	0.5
Age (years)	0.99	0.97, 1.02	0.6
Sex (ref: male)			
female	1.32	0.59, 3.05	0.5
Baseline headache days	1.00	0.91, 1.09	>0.9
Chronic migraine			
yes	0.29	0.07, 1.08	0.072
Daily headache			
yes	0.22	0.03, 1.22	0.11
Medication overuse headache			
yes	4.09	1.55, 12.3	**0.007**
Psychiatric comorbidity			
no	1.28	0.56, 3.12	0.6
Concomitant preventive medication			
yes	0.89	0.45, 1.77	0.7
Erenumab dose (ref: 140 mg)			
70 mg	1.02	0.39, 2.74	>0.9
Headache diary available			
Medical documentation only	0.72	0.33, 1.55	0.4

*Abbreviations*: MHD: Monthly headache days, *OR*: Odds ratio, *CI*: Confidence interval

^1^*OR* Odds Ratio, *CI* Confidence Interval

To assess whether modelling previous preventive treatment exposure as a continuous variable altered the findings, an additional multivariate logistic regression replacing treatment group with the number of previous preventive treatment failures was performed. This model showed comparable model fit and likewise demonstrated no association between the number of previous preventive treatment failures and treatment response (see Supplementary Table S1 A). Inclusion of migraine duration and aura status in complete-case analyses did not materially change the effect estimates (see Supplementary Table S1 B).

In exploratory analyses, LASSO variable selection retained chronic migraine, daily headache, MOH, sex, and baseline headache frequency as relevant candidate predictors. A subsequent multivariate logistic regression including these variables yielded results consistent with the primary analysis, with no significant association between treatment group (SPT/MPT) and treatment response (OR 1.20, 95% CI 0.58–2.45; *p* = 0.60) (see Supplementary Table S2).

As a further sensitivity analysis, IPTW based on the propensity score was performed. Stabilised weights ranged from 0.32 to 5.62 in the MPT group and from 0.72 to 4.86 in the SPT group. No weight truncation was applied, as no excessively large weights were observed. The effective sample size after weighting was 89.1 for MPT and 20.6 for SPT. Weighted logistic regression models were fitted using the survey package with robust (sandwich) variance estimation.

Covariate balance was reassessed using an absolute SMD threshold of < 0.10. Although weighting reduced imbalance for several covariates, residual imbalance remained for some variables (see Supplementary Figure S2). These covariates were therefore additionally included in the weighted outcome model to provide a doubly robust estimation. No significant association between treatment group (SPT vs. MPT) and treatment response was observed in the weighted analysis (OR 0.79, 95% CI 0.29–2.12; *p* = 0.6; see Supplementary Table S3).

In a subgroup analysis restricted to patients with available headache diary data (SPT: *n* = 11; MPT: *n* = 101), SPT was not associated with a higher likelihood of achieving a ≥ 50% reduction in monthly headache days compared with MPT (OR 0.66, 95% CI 0.07–5.18, *p* = 0.70). Although the point estimate differed from that of the primary analysis, the confidence interval was wide and included 1, reflecting the limited precision of the estimate due to the small subgroup size, particularly in the SPT group. The overall interpretation of the findings remained unchanged. Responder rates and detailed model results are provided in Supplementary Table S4.

Overall, the proportion of patients experiencing adverse events was comparable between the SPT and MPT groups. Detailed results regarding adverse events and changes in migraine characteristics are provided in the Supplementary Material (“Migraine Characteristics and Adverse Events”).

## Discussion

In this real-world study, two cohorts of patients receiving erenumab for migraine prevention in Germany were investigated: one after multiple prior preventive treatment failures (MPT) and another after a maximum of one prior preventive treatment failure (SPT), largely enabled by a change in reimbursement policy. Our multivariate model showed no significant difference in achieving ≥ 50% reduction in MHD between patients in the SPT and MPT groups. In the propensity score weighting sensitivity analysis, SPT was not associated with higher odds of achieving a ≥ 50% reduction in MHD compared with MPT. Although weighting reduced baseline imbalances, residual imbalance, limited treatment overlap and the resulting reduction in effective sample size may have affected the precision of the estimates. Although point estimates varied across models, all confidence intervals included the null value (OR = 1), and no statistically significant association was observed. Given the width of the confidence intervals and the limited precision of the estimates, however, the absence of a statistically significant association should not be interpreted as evidence of no effect. Thus, a potential effect in either direction can neither be reliably established nor excluded based on the present data.

The multivariate model identified MOH as a factor associated with a higher likelihood of treatment response (OR 4.09, 95% CI 1.55–12.3; *p* = 0.007). However, this finding should be interpreted with caution given the wide confidence interval and the relatively small cohort. Although we have previously shown in a real-world setting that MOH is not consistent associated with a poorer response to CGRP mAbs [9], the present finding should be interpreted with caution, as it may reflect cohort-specific effects rather than a true biological association. The uncertainty surrounding this result is further emphasized by the broad confidence interval.

Cohort specific differences becomes particularly apparent when comparing our findings with those of Hong et al., who also conducted a real-world study in Germany investigating the impact of the reimbursement policy change for CGRP mAbs [5]. In the referenced study, the 50% responder rate for MMD was 40.6% in the old policy group (*n* = 146, almost equivalent to the MPT group in our study) and 60% in the new policy group (*n* = 63, almost equivalent to the SPT group). In contrast, our study showed comparable 50% responder rates between the two groups (45.7% vs. 50.0%). Similarly, for MHD, our results demonstrated comparable 50% responder rates in MPT and SPT patients (30.7% vs. 35.7%), whereas Hong et al. reported 37.7% for the old policy group and substantially higher (63.5%) for the new policy group. Overall, the referenced study found markedly higher responder rates in the new policy cohort, while our findings contradict these assertions and show no differences between the MPT and SPT groups.

Hong et al. employed a slightly different cohort definition compared with ours. They defined the “old policy” group as patients who had tried and failed, or had contraindications to all first-line prophylactic medication classes, whereas the “new policy” group included patients after at least one prophylactic medication trial. In rare cases, patients may have no alternative to a single non-specific treatment, which could result in contraindications and appropriate classification as “old policy,” even in the context of “SPT” of preventive therapy. We therefore categorized our groups solely based on the number of preventive treatments received. Nevertheless, differences in cohort definitions may potentially reduce comparability with the referenced study.

The differing results across our studies highlight the challenges of drawing general conclusions regarding the optimal timing of treatment initiation and predictors of treatment response under real-world conditions. In contrast to the assumptions by Hong et al., our findings do not support the hypothesis that initiating erenumab after failure of only one prior preventive therapy results in superior short-term treatment response. These observations suggest that factors beyond the number of previous preventive treatment failures influence therapeutic response.

In the APPRAISE trial, erenumab was compared with non-specific oral migraine preventive medications (NOMPMs) in patients with EM. The primary outcome was the proportion of patients who both remained on their initially assigned treatment for one year and achieved at least a 50% reduction in MMD at month 12. Early treatment with erenumab was associated with substantially greater efficacy, safety, and adherence. Significantly more patients receiving erenumab achieved the primary endpoint compared with those treated with NOMPMs (56.2% vs. 16.8%), indicating a beneficial effect of early treatment initiation [10]. However, an important limitation of the APPRAISE study is that it included only patients who had already received at least one prior oral preventive therapy. It is well established that adherence to oral preventive therapies is low [11], further increasing the likelihood that individuals with poor tolerability or insufficient effectiveness of conventional preventives are overrepresented in the study. Individuals who respond well to and tolerate the first conventional preventive treatment were therefore not represented. This reduces the generalisability of the findings. Treatment naïve patients might exhibit substantially better outcomes and would likely remain on oral therapy for as long as it is effective and well tolerated, meaning they would potentially never, or only much later, reach a point at which switching to a CGRP mAb becomes necessary. At the same time, lack of response to preventive therapies is common in clinical practice and has been reported to affect up to approximately 50% of migraine patients, suggesting that the study population still reflects a substantial proportion of individuals seen in routine care [12].

Another study by Caronna et al., conducted as a large, multicentre, real-world investigation, examined the effectiveness of CGRP mAb in preventing migraine [13]. The analysis showed a negative linear association between the number of MMD at baseline and the probability of a positive treatment response. Patients who began therapy at lower attack frequencies were markedly more likely to achieve a 50% reduction in MHD. In contrast, response rates declined significantly in patients with high MMD at baseline. According to the authors, these findings indicate that initiating CGRP mAb earlier, before migraine burden reaches its highest levels, enhances the probability of clinically meaningful benefit.

In the study by Caronna et al., the majority of patients were refractory to conventional preventive medications. This finding may suggest that early treatment with CGRP mAb could be beneficial in preventing chronification. Although our three-month follow-up precludes conclusions regarding the long-term risk of migraine chronification, our adjusted multivariate model demonstrated that the number of prior preventive treatment failures, reflected by allocation to the MPT or SPT group, was not independently associated with short-term treatment response. Thus, our data do not support the notion that initiation of erenumab at the beginning of preventive treatment per se is necessary to achieve treatment effectiveness.

However, treatment should ideally be implemented before substantial disease progression or severe chronification occurs. We would therefore interpret our findings and the findings of Caronna et al. as supporting the need for an initiation of an effective preventive therapy. This may involve conventional preventive treatment when effective and well tolerated, with CGRP mAbs potentially considered as an early option in selected patients in whom refractoriness to conventional oral therapies may become apparent. Although the gradual changes in reimbursement regulations for erenumab in Germany provided a unique opportunity to compare earlier versus later treatment initiation according to the number of prior preventive treatment failures, our findings should be interpreted with caution because of the substantially smaller sample size compared with the study by Caronna et al. (196 vs. 4,963 patients).

Nevertheless, a meta-analysis of randomised erenumab trials found no significant subgroup differences between EM and CM with regard to MMD reduction or ≥ 50% responder rates. These findings suggest that migraine type per se does not substantially influence the therapeutic effect of erenumab [14], which is consistent with the findings of our analysis. Considering the relatively small EM and CM subgroup sizes in our cohort, which limit direct comparisons of response rates, treatment effects and responder rates among CM patients were nevertheless virtually identical between the MPT and SPT groups. Our findings did not provide clear evidence that initiation of erenumab after multiple preventive treatment failures was associated with reduced effectiveness in patients with CM and were therefore broadly consistent with the findings of the aforementioned meta-analysis.

Socioeconomic aspects should also be considered. In Germany, changes in reimbursement regulations have enabled earlier initiation of erenumab therapy. As CGRP-targeting therapies are substantially more expensive than conventional preventive medications, their broader use raises important health economic considerations. However, previous studies have suggested that, particularly in patients with CM who respond to CGRP mAbs, reductions in indirect costs, such as productivity loss, work absenteeism, and healthcare utilization, may outweigh the higher drug acquisition costs [15–17]. Whether this also applies to the broader migraine population remains uncertain. These considerations further emphasize the need to identify patients at high risk of migraine chronification who are most likely to benefit from early treatment with CGRP-targeting therapies. Reliable clinical and biological biomarkers capable of identifying patients at greatest risk of chronification and accurately predicting treatment response could facilitate personalised treatment decisions, enabling early initiation of the most appropriate preventive therapy while improving both clinical outcomes and the cost-effectiveness of migraine care.

In our study, patients were stratified according to the number of prior preventive treatment failures, comparing those with no more than one prior treatment failure with those who had experienced multiple prior preventive treatment failures. However, defining disease stage in migraine remains methodologically challenging, as the disorder does not follow a predictable linear progression from episodic to chronic forms. Symptom duration alone is therefore insufficient to determine disease stage. In this context, the number of prior preventive treatment attempts may serve as a pragmatic surrogate for cumulative disease burden and therapeutic refractoriness. Nevertheless, this variable cannot be interpreted as a direct measure of disease stage. While multiple prior therapies may reflect prolonged suffering or inadequate treatment response, this association may be influenced by differences in healthcare access, referral pathways, and treatment strategies, which can also affect the timing of even the first initiation of conventional preventive therapies. Accordingly, the number of prior therapies represents an imperfect but feasible proxy within the constraints of real-world data. It should also be noted that the distinction between “early” (after SPT) or “late” (after MPT) erenumab initiation in our study was not based on the duration of preceding preventive therapies. Due to the retrospective design, detailed information on the exact duration of each prior treatment was not consistently available. Consequently, potential group differences in cumulative preventive treatment exposure before erenumab initiation could not be assessed and warrant evaluation in future prospective studies.

Several limitations should be considered when interpreting these findings. First, this was a non-randomised real-world study, and residual confounding due to unmeasured factors cannot be excluded despite multivariable adjustment and propensity score weighting. Second, the relatively small sample size and limited number of treatment responders resulted in a modest EPV ratio and wide confidence intervals, thereby limiting the precision and stability of the regression estimates. Within these constraints, we did not detect a clear association between prior preventive treatment burden (SPT vs. MPT) and the likelihood of achieving a ≥ 50% reduction in MHD. However, the limited precision of the estimates precludes interpreting these findings as evidence for the absence of an association. Furthermore, the study was designed to evaluate differences in overall treatment response between the SPT and MPT groups and was not sufficiently powered to reliably assess treatment-effect modification. Formal interaction analyses were therefore not performed. Consequently, whether the association between prior preventive treatment burden and treatment response varies according to factors such as chronic migraine status or baseline headache frequency remains uncertain and should be evaluated in larger studies. Third, missing data for migraine duration and aura status precluded their inclusion in the primary model. Fourth, the propensity score-weighted analysis showed limited overlap between treatment groups, with substantial weight variability and a reduced effective sample size, which may affect the robustness of the weighted estimates. Fifth, treatment response was assessed after only three months of follow-up, and the findings may therefore not reflect the longer-term effectiveness of erenumab. Sixth, our classification of patients according to the number of prior preventive treatment failures represents a pragmatic measure of previous treatment exposure but does not capture potentially relevant factors such as treatment duration, adequacy of dosing, adherence, or the reasons for discontinuation of previous preventive therapies. As these variables were not systematically documented in this retrospective real-world cohort, residual heterogeneity and unmeasured confounding cannot be excluded. Seventh, the exclusion of patients with insufficient follow-up data, including some who may have discontinued treatment before the 3-month assessment, may have introduced attrition bias if patients with missing follow-up differed systematically from those who completed the assessment. This may have influenced the estimated treatment effectiveness and could have resulted in an overestimation if patients with less favourable outcomes were more likely to discontinue treatment or have missing follow-up data. Eighth, the two treatment groups were recruited during substantially different calendar periods, reflecting changes in reimbursement criteria over the study period. Although the year of treatment initiation is presented in Table 1, the limited temporal overlap between the groups precluded reliable separation of treatment-group effects from secular changes in clinical practice, patient selection, and prescribing patterns. Residual confounding by unmeasured temporal factors therefore cannot be excluded.

Finally, differences in data collection methods, including headache diaries and routine clinical documentation, may have affected the study findings. Although this variable was not independently associated with treatment response in our multivariate model, the potential for information bias resulting from heterogeneous data collection should be acknowledged as a limitation of the study.

In principle, randomised controlled trials would be the preferred study design to determine the optimal timing of treatment initiation. However, a trial designed to definitively address this question would inevitably require delaying effective treatment in some patients, thereby potentially allowing disease progression or migraine chronification, which would not be ethically justifiable. Consequently, only certain aspects of this question can be investigated under controlled conditions, making evidence from well-designed real-world studies indispensable. Further longitudinal analyses, particularly in real-world settings, are therefore warranted to better define the optimal timing of CGRP mAb therapy.

## Conclusion

In this real-world study, we found no clear evidence that initiating erenumab after no more than one prior treatment failure resulted in different short-term outcomes compared with initiation after multiple preventive treatment failures. Both the SPT and MPT groups showed substantial reductions in MHD, MMD, and AMD after three months of treatment. The magnitude of improvement and responder rates were largely comparable between groups, suggesting that short-term treatment effectiveness was independent of the number of prior preventive treatment failures. Comparison with a similar single-center real-world study revealed marked cohort-specific effects, highlighting the challenges of drawing generalised conclusions from observational data. These findings underscore that treatment response may be influenced by factors beyond previous preventive treatment failures and emphasise the need for larger, multicenter studies with standardised data collection. Nevertheless, the possibility that initiating treatment after fewer prior preventive treatment failures may confer additional long-term benefits remains an important hypothesis and warrants further investigation in prospective longitudinal studies, both in controlled clinical trials and real-world settings, to better define the optimal timing of CGRP mAb treatment initiation.

## Supplementary Information


Supplementary Material 1. Figure S1.



Supplementary Material 2. Figure S2.



Supplementary Material 3. Table S1.



Supplementary Material 4. Table S2.



Supplementary Material 5. Table S3.



Supplementary Material 6. Table S4.



Supplementary Material 7. Migraine Characteristics and Adverse Events.



Supplementary Material 8. Q0mo.



Supplementary Material 9. Q3mo.


## Data Availability

The datasets used and/or analysed during the current study are available from the corresponding author on reasonable request.

## Abbreviations

AMD: Monthly Acute Medication Days
ATE: Average treatment effect
CM: Chronic Migraine
CGRP: Calcitonin Gene-Related Peptide
CI: Confidence Interval
EM: Episodic Migraine
EMA: European Medicine Agency
IQR: Interquartile Range
IPTW: Inverse Probability of Treatment Weighting
mAb: Monoclonal Antibody
MHD: Monthly Headache Days
MMD: Monthly Migraine Days
MOH: Medication Overuse Headache
MPT: Multiple prior preventive therapies
NOMPM: Non-specific Oral Migraine Preventive Medications
OR: Odds Ratio
SPT: Single prior preventive therapy
