# Supplementary material for: Real-world short-term effectiveness of erenumab after single versus multiple prior preventive treatment failures
Source: BMC Neurol. 2026 Aug 28;26:550. doi: 10.1186/s12883-026-05311-8 (PMC13523192; doi:10.1186/s12883-026-05311-8)
Supplement: Supplementary file 1 [file 12883_2026_5311_MOESM1_ESM.docx]

**Figure S1: Baseline MHD, MMD and AMD of SPT and MPT group.** (Abbreviations: AMD: Acute medication days, SPT: Single prior preventive therapy, MPT: Multiple prior preventive therapies, MHD: monthly headache days, MMD: monthly migraine days).
