# Supplementary material for: Real-world short-term effectiveness of erenumab after single versus multiple prior preventive treatment failures
Source: BMC Neurol. 2026 Aug 28;26:550. doi: 10.1186/s12883-026-05311-8 (PMC13523192; doi:10.1186/s12883-026-05311-8)
Supplement: Supplementary file 2 [file 12883_2026_5311_MOESM2_ESM.docx]

**Supplementary Material Fig S2: Love Plot**

To further address potential confounding inherent to this observational study, inverse probability of treatment weighting (IPTW) based on the propensity score was performed as a sensitivity analysis.

The propensity score was estimated using the same baseline covariates included in the primary multivariable model. Covariate balance before and after weighting was assessed using standardized mean differences (SMDs), with an SMD < 0.1 considered indicative of adequate balance, whereas an SMD $\geq$ 0.1 indicated relevant residual imbalance.
