# Supplementary material for: Real-world short-term effectiveness of erenumab after single versus multiple prior preventive treatment failures
Source: BMC Neurol. 2026 Aug 28;26:550. doi: 10.1186/s12883-026-05311-8 (PMC13523192; doi:10.1186/s12883-026-05311-8)
Supplement: Supplementary file 3 [file 12883_2026_5311_MOESM3_ESM.docx]

**Supplementary Table S1.** Adjusted multivariate logistic regression analysis with achievement of a ≥50% reduction in monthly headache days (MHD) as the dependent variable.

**(A)** Multivariate model including all prespecified covariates of the primary analysis, with the number of prior preventive treatment failures entered as a continuous variable instead of treatment group (SPT vs. MPT). The responder rates, defined as a ≥50% reduction in MHD, in the population included in this analysis were identical to those observed in the overall cohort, as reported in Table 3 (MPT: 43/140 (30.7%); SPT: 20/56 (35.7%)).

| **Characteristic** | **OR** | **95% CI** | **p-value** |
| --- | --- | --- | --- |
| Number of pre-treatments | 1.04 | 0.84, 1.30 | 0.7 |
| Age (years) | 0.99 | 0.96, 1.02 | 0.6 |
| Sex (ref: male) |  |  |  |
| female | 1.37 | 0.61, 3.15 | 0.5 |
| Baseline headache days | 1.00 | 0.91, 1.09 | >0.9 |
| Chronic migraine |  |  |  |
| yes | 0.28 | 0.07, 1.04 | 0.064 |
| Daily headache |  |  |  |
| yes | 0.23 | 0.03, 1.25 | 0.11 |
| Medication overuse headache |  |  |  |
| yes | 4.17 | 1.58, 12.5 | 0.006 |
| Psychiatric comorbidity |  |  |  |
| no | 1.24 | 0.54, 3.02 | 0.6 |
| Concomitant preventive medication |  |  |  |
| yes | 0.87 | 0.44, 1.71 | 0.7 |
| Erenumab dose (ref: 140mg) |  |  |  |
| 70 | 0.79 | 0.29, 2.09 | 0.6 |
| Headache diary available |  |  |  |
| Medical documentation only | 0.79 | 0.37, 1.66 | 0.5 |
| CI: Confidence Interval, MPT: Multiple prior preventive therapies, OR: Odds Ratio, SPT: Single prior preventive therapy | | | |

**(B)** Multivariate model including all prespecified covariates, with treatment group (single prior preventive treatment failure [SPT] vs. multiple prior preventive treatment failures [MPT]) as the primary explanatory variable.

| **Characteristic** | **OR** | **95% CI** | **p-value** |
| --- | --- | --- | --- |
| Treatment group (SPT vs MPT) |  |  |  |
| MPT | — | — |  |
| SPT | 1.46 | 0.52, 4.12 | 0.5 |
| Age (years) | 0.98 | 0.94, 1.02 | 0.3 |
| Sex (ref: male) |  |  |  |
| female | 1.30 | 0.54, 3.24 | 0.6 |
| Baseline headache days | 1.04 | 0.94, 1.14 | 0.5 |
| Chronic migraine |  |  |  |
| yes | 0.26 | 0.06, 1.11 | 0.076 |
| Daily headache |  |  |  |
| yes | 0.09 | 0.00, 0.67 | 0.041 |
| Medication overuse headache |  |  |  |
| yes | 3.03 | 1.07, 9.55 | 0.044 |
| Psychiatric comorbidity |  |  |  |
| no | 1.41 | 0.56, 3.84 | 0.5 |
| Concomitant preventive medication |  |  |  |
| yes | 0.97 | 0.46, 2.07 | >0.9 |
| Migraine duration | 1.03 | 0.99, 1.07 | 0.14 |
| Erenumab dose (ref: 140mg) |  |  |  |
| 70mg | 1.07 | 0.38, 3.05 | >0.9 |
| Aura |  |  |  |
| no | 1.89 | 0.92, 3.96 | 0.086 |
| NA | 0.90 | 0.04, 9.00 | >0.9 |
| Headache diary available |  |  |  |
| Medical documentation only | 0.98 | 0.42, 2.25 | >0.9 |
| CI: Confidence Interval, MPT: Multiple prior preventive therapies, NA: Not available, OR: Odds Ratio, SPT: Single prior preventive therapy | | | |

|  | **SPT** | **MPT** | **Total** |
| --- | --- | --- | --- |
| **Responder** | 18 (35%) | 38 (31%) | 56 (32%) |
| **Non-Responder** | 34 (65%) | 84 (69%) | 118 (68%) |
| **Total** | 52 | 122 | 174 |

This model included all available covariates. Due to missing data for one or more variables, 22 of the 196 patients were excluded, resulting in a complete-case population of 174 patients. The distribution of responders and non-responders (≥50% reduction in monthly headache days (MHD)) according to treatment group is shown in the table. (SPT: single prior preventive treatment failure, MPT: multiple prior preventive treatment failures):
