# Supplementary material for: Real-world short-term effectiveness of erenumab after single versus multiple prior preventive treatment failures
Source: BMC Neurol. 2026 Aug 28;26:550. doi: 10.1186/s12883-026-05311-8 (PMC13523192; doi:10.1186/s12883-026-05311-8)
Supplement: Supplementary file 4 [file 12883_2026_5311_MOESM4_ESM.docx]

**Supplementary Material Table S2: LASSO-penalized logistic regression**

| **Characteristic** | **OR** | **95% CI** | **p-value** |
| --- | --- | --- | --- |
| Treatment group (SPT vs MPT) |  |  |  |
| MPT | — | — |  |
| SPT | 1.20 | 0.58, 2.45 | 0.6 |
| Sex (ref: male) |  |  |  |
| female | 1.32 | 0.61, 2.97 | 0.5 |
| Baseline headache days | 1.0 | 0.91, 1.09 | >0.9 |
| Chronic migraine |  |  |  |
| yes | 0.30 | 0.08, 1.11 | 0.082 |
| Daily headache |  |  |  |
| yes | 0.23 | 0.03, 1.21 | 0.11 |
| Medication overuse headache |  |  |  |
| yes | 3.93 | 1.52, 11.6 | 0.007 |
| CI: Confidence Interval, MPT: Multiple prior preventive therapies, OR: Odds Ratio, SPT: Single prior preventive therapy | | | |

As an exploratory analysis, a LASSO-penalized logistic regression was performed. A 10-fold cross-validation procedure was used to determine the optimal penalty parameter. Variables with non-zero coefficients after penalization were considered candidate predictors of treatment response.

The LASSO analysis was intended to explore predictor importance and support model parsimony rather than to replace the prespecified multivariable regression model. Because treatment group represented the primary exposure of interest, it was retained in all inferential models regardless of penalized variable selection results.

The responder rates, defined as a ≥50% reduction in MHD, in the population included in this analysis were identical to those observed in the overall cohort, as reported in Table 3 (MPT: 43/140 (30.7%); SPT: 20/56 (35.7%)).
