# Supplementary material for: Real-world short-term effectiveness of erenumab after single versus multiple prior preventive treatment failures
Source: BMC Neurol. 2026 Aug 28;26:550. doi: 10.1186/s12883-026-05311-8 (PMC13523192; doi:10.1186/s12883-026-05311-8)
Supplement: Supplementary file 5 [file 12883_2026_5311_MOESM5_ESM.docx]

**Supplementary Table S3: Weighted Logistic Regression After Propensity Score Weighting**

A weighted logistic regression model was fitted using the propensity score weights. The responder rates, defined as a ≥50% reduction in MHD, in the population included in this analysis were identical to those observed in the overall cohort, as reported in Table 3 (MPT: 43/140 (30.7%); SPT: 20/56 (35.7%))

| **Characteristic** | **OR** | **95% CI** | **p-value** |
| --- | --- | --- | --- |
| Treatment group (SPT vs MPT) |  |  |  |
| MPT | — | — |  |
| SPT | 0.79 | 0.19, 2.12 | 0.6 |
| Age | 0.99 | 0.96, 1.03 | 0.7 |
| Baseline headache days | 0.95 | 0.90, 1.00 | 0.042 |
| Headache diary available |  |  |  |
| Medical documentation only | 0.82 | 0.37, 1.83 | 0.6 |
| CI = Confidence Interval, MPT: Multiple prior preventive therapies, OR = Odds Ratio, SPT: Single prior preventive therapy | | | |
