# Supplementary material for: Real-world short-term effectiveness of erenumab after single versus multiple prior preventive treatment failures
Source: BMC Neurol. 2026 Aug 28;26:550. doi: 10.1186/s12883-026-05311-8 (PMC13523192; doi:10.1186/s12883-026-05311-8)
Supplement: Supplementary file 6 [file 12883_2026_5311_MOESM6_ESM.docx]

**Supplementary Table S4.** Subgroup analysis. This model included only patients for whom headache outcome data were available from a headache diary. Adjusted multivariate logistic regression analysis with achievement of a ≥50% reduction in monthly headache days (MHD) as the dependent variable.

| **Characteristic** | **OR** | **95% CI** | **p-value** |
| --- | --- | --- | --- |
| Treatment group (SPT vs MPT) |  |  |  |
| MPT | — | — |  |
| SPT | 0.66 | 0.07, 5.18 | 0.7 |
| Age (years) | 1.01 | 0.97, 1.05 | 0.7 |
| Sex (ref: male) |  |  |  |
| female | 1.13 | 0.36, 3.62 | 0.8 |
| Baseline headache days | 0.90 | 0.79, 1.02 | 0.10 |
| Chronic migraine |  |  |  |
| yes | 0.37 | 0.06, 1.97 | 0.3 |
| Daily headache |  |  |  |
| yes | 0.47 | 0.02, 5.27 | 0.6 |
| Medication overuse headache |  |  |  |
| yes | 8.44 | 2.30, 42.3 | 0.003 |
| Psychiatric comorbidity |  |  |  |
| no | 0.85 | 0.23, 3.26 | 0.8 |
| Concomitant preventive medication |  |  |  |
| yes | 1.05 | 0.41, 2.70 | >0.9 |
| Erenumab dose (ref: 140mg) |  |  |  |
| 70mg | 0.49 | 0.07, 3.42 | 0.5 |
| CI: Confidence Interval, MPT: Multiple prior preventive therapies, OR: Odds Ratio, SPT: Single prior preventive therapy | | | |

The distribution of responders and non-responders (≥50% reduction in monthly headache days (MHD)) according to treatment group is shown in the table. (SPT: single prior preventive treatment failure, MPT: multiple prior preventive treatment failures):

|  | **SPT** | **MPT** | **Total** |
| --- | --- | --- | --- |
| **Responder** | 4 (36%) | 33 (33%) | 37 (33%) |
| **Non-Responder** | 7 (64%) | 68 (67%) | 75 (67%) |
| **Total** | 11 | 101 | 112 |
