# Supplementary material for: Real-world short-term effectiveness of erenumab after single versus multiple prior preventive treatment failures
Source: BMC Neurol. 2026 Aug 28;26:550. doi: 10.1186/s12883-026-05311-8 (PMC13523192; doi:10.1186/s12883-026-05311-8)
Supplement: Supplementary file 7 [file 12883_2026_5311_MOESM7_ESM.docx]

**Migraine Characteristics**

Changes in migraine characteristics are summarised in Supplementary Figure S2. Migraine intensity showed the greatest improvement, with more than 60% of patients in both groups reporting reduced attack intensity. More than half of the patients also reported shorter attack duration. One patient in each group experienced complete freedom from migraine attacks during the observation period. Most patients reported no substantial changes in associated symptoms, including photophobia, phonophobia, dizziness, nausea, need for rest, aura, or response to acute medication after three months of treatment. With regard to associated symptoms such as vertigo, photophobia, phonophobia, and the need for rest, the majority of patients in both groups reported no relevant change. Similarly, no differences in the response to acute medication were reported after three months of therapy.

**Figure S2:** **Patient-reported subjective changes after three months of therapy in the SPT and MPT group**. For each category, patients reported whether their condition had improved, worsened, remained unchanged, or was not present (not existing). (Abbreviations: SPT: Single prior preventive therapy, MPTI: Multiple prior preventive therapies).

**Adverse Events**

Adverse events were documented to assess the tolerability of the treatment in both groups. Among SPT patients, 35.7% (n=20/56, NA: 3) experienced adverse events. Within the MPT group, adverse events were reported by 34.3% (n=48/140, NA: 1). A comparison of the adverse events revealed that constipation was the most frequently reported side effect in both groups (MPT: 23 cases vs. SPT: 4 cases). Pruritus (4 vs. 0), sleep disorders (5 vs. 1) and dizziness (4 vs. 1) were likewise more commonly observed in MPT. Furthermore, hair loss was reported only in the MPT group (1 vs. 0), whereas neck pain and flushing were documented exclusively in the SPT group (0 vs. 1, see Supplementary Table S4).

|  | **MPT (n=140)** | **SPT (n=56)** | **p-value** |
| --- | --- | --- | --- |
|  |  |  |  |
| Constipation | 23 (15.5%) | 4 (7.1%) | 0.14 |
| Pruritus | 4 (2.7%) | 0 (0%) | 0.58 |
| Sleep disorders | 5 (3.6%) | 1 (1.8%) | 0.66 |
| Tiredness | 4 (2.7%) | 0 (0%) | 0.58 |
| Dizziness | 4 (2.7%) | 1 (1.8%) | 1.0 |

**Table S4: Adverse events in SPT and MPT group.**

(Abbreviations: SPT: Single prior preventive therapy, MPT: Multiple prior preventive therapies).
