# Supplementary material for: Real-world short-term effectiveness of erenumab after single versus multiple prior preventive treatment failures
Source: BMC Neurol. 2026 Aug 28;26:550. doi: 10.1186/s12883-026-05311-8 (PMC13523192; doi:10.1186/s12883-026-05311-8)
Supplement: Supplementary file 8 [file 12883_2026_5311_MOESM8_ESM.docx]

# Pre-Antibody Therapy Questionnaire

**Headache Frequency (last 3 months):**

| Total headache days |  |
| --- | --- |
| Migraine days ONLY |  |
| Days with acute medication |  |
| Days missed at work |  |

**Effect of Acute Medication**

| **Medication** | **Effect (Yes / No / Partial)** |
| --- | --- |
|  |  |
|  |  |
|  |  |
|  |  |

**Preventive Medications Tried**

Have you tried preventive medications?

☐ Yes ☐ No

| **Ever taken?** | | **If you answered “yes”** | | |
| --- | --- | --- | --- | --- |
| **Medication** | **(Yes / No / Contraindication*)** | **Currently taking (Yes/No)** | **Period (from–to)** | **Reason (ineffective / side effects)** |
| **Beta-blockers:**  Propranolol  OR/AND  Metoprolol  OR/AND  Others: |  |  |  |  |
| **Topiramate** |  |  |  |  |
| **Flunarizine** |  |  |  |  |
| **Amitriptyline** |  |  |  |  |
| **Valproate** |  |  |  |  |
| **OnabotulinumtoxinA** |  |  |  |  |

*Were you unable to take the medication for other reasons?

**Other medications taken/tried? Have you already tried antibody therapy?**

| Medication | Are you still taking the medication? | If no: reason for discontinuation (side effects / insufficient effect?) |
| --- | --- | --- |
|  |  |  |
|  |  |  |
|  |  |  |
|  |  |  |

**Non-Pharmacological Treatments**

| **Treatment** | **Frequency** | **Since when** |
| --- | --- | --- |
| **Endurance sports** |  |  |
| **Relaxation techniques** |  |  |
| **Homeopathy** |  |  |
| **Osteopathy** |  |  |
| **Acupuncture** |  |  |

**Medical History**

Other conditions:

_____________________________________________________________________________________________________________________________________________________________________________________________________________________________________________________________________________________________________________________________________________________________________________________________________________________________________________________________________________________________________________________________________________

**Other Medications**

| **Active substance** | **Duration** |
| --- | --- |
|  |  |
|  |  |
|  |  |
|  |  |
|  |  |

**Patient Declaration**

I hereby confirm that the information I have provided is accurate. I have also received detailed information about the active substance, the form of administration, as well as possible side effects and contraindications, in particular regarding the potential teratogenicity of the medication.

Place, Date: ____________________	Signature: ____________________

**To be completed by the physician**

**Diagnosis:**

☐ Episodic migraine without aura
☐ Episodic migraine with/without aura
☐ Menstrual migraine
☐ Vestibular migraine
☐ Chronic migraine without aura
☐ Chronic migraine with/without aura

☐ Medication overuse headache

**CGRP receptor antibody (Erenumab) eligibility**

☐ Yes ☐ No

**Selected dosage:**

☐ 70 mg ☐ 140 mg

**Previous antibody therapy?**

☐ Yes ☐ No

If yes:

- Which one? ______________________________________
- Duration: ________________________________________
- Dosage: _________________________________________
- Reason for switching: ______________________________

**Headache diary available and reviewed?**

☐ Yes ☐ No

The patient has been informed about known side effects of Erenumab, especially allergic reactions at the injection site and constipation. Female patients have been advised to avoid pregnancy during treatment, as no data on teratogenicity is currently available. The first administration was carried out at the West German Headache Center under supervision of a headache nurse.

Place, Date: __________________	Physician Signature:________________
