# Supplementary material for: Real-world short-term effectiveness of erenumab after single versus multiple prior preventive treatment failures
Source: BMC Neurol. 2026 Aug 28;26:550. doi: 10.1186/s12883-026-05311-8 (PMC13523192; doi:10.1186/s12883-026-05311-8)
Supplement: Supplementary file 9 [file 12883_2026_5311_MOESM9_ESM.docx]

**3-Month Antibody Therapy Follow-Up Questionnaire**

**Patient Questionnaire**

**Information for the last 3 months under antibody therapy**

| Total headache days (last 3 months) |  |
| --- | --- |
| Migraine days only (last 3 months) |  |
| Days with acute medication (last 3 months) |  |
| Days missed at work (last 3 months) |  |

**Therapy Details**

| Number of injections |  |
| --- | --- |
| Dosage (mg) |  |

**Additional Factors**

Stress factors (illness, job change, etc.):

Lifestyle changes (sports, relaxation, etc.):

**Current Preventive Medication (excluding antibodies)**

| **Active substance** | **Dose** | **Duration** |
| --- | --- | --- |
|  |  |  |
|  |  |  |
|  |  |  |

**Effect of Acute Medication**

| **Medication** | **Effect (Yes / No / Partial)** |
| --- | --- |
|  |  |
|  |  |
|  |  |

**Change in headaches over the last 3 months under therapy (please tick):**

|  | **Improved** | **Worse** | **Unchanged** | **Not present*** |
| --- | --- | --- | --- | --- |
| **Intensity** |  |  |  |  |
| **Duration** |  |  |  |  |
| **Response to acute medication** |  |  |  |  |
| **Aura** |  |  |  |  |
| **Vertigo** |  |  |  |  |
| **Nausea/Vomiting** |  |  |  |  |
| **Light/Noise sensitivity** |  |  |  |  |
| **Need for rest** |  |  |  |  |

*If you do not have this symptom/situation, please tick this box.

**Side Effects:**

☐ Yes ☐ No

If yes: ______________________________________

**Questions and comments:**

**Overall satisfaction with the therapy (please tick):**

☐ Very dissatisfied ☐ Dissatisfied ☐ Moderate ☐ Satisfied ☐ Very satisfied

**Physician Section**

Headache diary reviewed?

☐ Yes ☐ No

Previous antibody therapy?

☐ Yes ☐ No

Procedure:

☐ Continue dosage

☐ Change dosage: __________

☐ Switch to: __________

☐ Discontinue therapy

**Date, Signature: _**___________________
